# Chronic tianeptine induces tolerance in analgesia and hyperlocomotion via mu-opioid receptor activation in mice

**DOI:** 10.3389/fpsyt.2023.1186397

**Published:** 2023-05-23

**Authors:** Florence Allain, Aliza T. Ehrlich, Michael McNicholas, Florence Gross, Weiya Ma, Brigitte L. Kieffer, Emmanuel Darcq

**Affiliations:** ^1^Douglas Hospital Research Center, Department of Psychiatry, McGill University, Montreal, QC, Canada; ^2^Centre de Recherche en Biomédecine de Strasbourg, INSERM, Université de Strasbourg, Strasbourg, France

**Keywords:** opioid, tail immersion, locomotor activity, place preference, mu-opioid receptor (MOR), tianeptine

## Abstract

**Introduction:**

Tianeptine is approved in some countries to treat depression and anxiety. In addition to its activity on serotonin and glutamate neurotransmission, tianeptine has been proven to be a mu-opioid receptor (MOR) agonist, but only a few preclinical studies have characterized the opioid-like behavioral effects of tianeptine.

**Methods:**

In this study, we tested tianeptine activity on G protein activation using the [S35] GTPγS binding assay in brain tissue from MOR+/+ and MOR−/− mice. Then, to determine whether tianeptine behavioral responses are MOR-dependent, we characterized the analgesic, locomotor, and rewarding responses of tianeptine in MOR+/+ and MOR−/− mice using tail immersion, hot plate, locomotor, and conditioned place preference tests.

**Results:**

Using the [S35] GTPγS binding assay, we found that tianeptine signaling is mediated by MOR in the brain with properties similar to those of DAMGO (a classic MOR agonist). Furthermore, we found that the MOR is necessary for tianeptine's analgesic (tail immersion and hot plate), locomotor, and rewarding (conditioned place preference) effects. Indeed, these behavioral effects could only be measured in MOR+/+ mice but not in MOR−/− mice. Additionally, chronic administration of tianeptine induced tolerance to its analgesic and hyperlocomotor effects.

**Discussion:**

These findings suggest that tianeptine's opioid-like effects require MOR and that chronic use could lead to tolerance.

## Introduction

Along with several other tricyclic compounds, tianeptine was one of the first atypical antidepressants to be patented in the early 1970s (1). The effectiveness of tianeptine as an antidepressant has been demonstrated in numerous clinical trials and is comparable to that of other antidepressants that have been clinically approved such as tricyclics and SSRIs (2, 3). Despite sharing a structural resemblance with classic tricyclic antidepressants, tianeptine seems to be mechanistically distinct. Indeed, the antidepressant action of tianeptine has been proposed as a selective enhancer of serotonin uptake (4) and then as a modulator of the glutamate system (5, 6). Then, Gassaway et al. (7) found that tianeptine is also a high-efficacy mu-opioid receptor (MOR) agonist.

However, only a few preclinical studies have characterized the opioid-like behavioral effects of tianeptine. Investigations in mice showed that agonist activity at MOR is required for tianeptine's antidepressant and other behavioral effects such as antinociception (8). The antidepressant effects of tianeptine were demonstrated to occur via GABAergic neurons (9). Specifically, they demonstrated that MOR expression in GABAergic cells, somatostatin-positive neurons, is required for the acute and chronic antidepressant-like responses to tianeptine using cell-type-specific MOR knockout mice. Additionally, they identified the ventral hippocampus as a potential site for antidepressant activity using a central infusion of tianeptine (9).

Furthermore, they showed that while fluoxetine promotes hippocampus neurogenesis, tianeptine does not (9). Interestingly, the combination of tianeptine and morphine twice daily for 6 days significantly reduced the development of tolerance to morphine-induced antinociception effects and abolished naloxone-precipitated withdrawal symptoms in C57BL/6 male mice (10). These findings indicate that tianeptine may be a potent inhibitor of both physical dependence and tolerance to morphine-induced antinociception in mice.

In this study, we first tested whether tianeptine signaling is mediated by MOR using the [S^35^] GTPγS assay in brain samples of mice lacking mu opioid receptors [MOR–/– mice (11)] and their MOR+/+ controls and compared signaling properties to those of DAMGO, a classic MOR agonist generally used as a reference. Then, we evaluated whether MOR is necessary for tianeptine-induced analgesia using tail immersion and hot plate tests in null mutant mice and their controls. We also investigated the locomotor and rewarding (using conditioned place preference) effects of tianeptine in MOR+/+ and MOR–/– mice. Finally, we examined the analgesic and hyperlocomotor effects of repeated administration of tianeptine.

## Materials and methods

### Mice

Breeding pairs of homozygous MOR–/– (11) or homozygous MOR+/+ mice allowed the generation of experimental male mice on a c57bl/6:sv129 (50:50) strain as originally described. For experiments involving chronic treatment with tianeptine, commercial mice (Jackson Laboratories) were ordered from the same c57bl/6:sv129 strain. The mice were housed 2–5 per cage under a 12-h light/dark cycle (lights on at 8:00 a.m.) in a temperature- and humidity-controlled room; they were 2–3 months old at the time of the behavioral tests. Water and food were available *ad libitum* until the end of the experiment. For each behavioral test, the mice were brought in 30 min before in the testing room for acclimatization. The Canadian Council of Animal Care and the Animal Care Committees of McGill University/Douglas Mental Health University Institute approved all experimental procedures.

### [^35^S] GTPγS binding assay

[^35^S] GTPγS binding assays were performed on membrane preparations from MOR+/+ mice or MOR–/– mice, following our previously published methods (12, 13). To assess [^35^S] GTPγS binding, brains were quickly removed after cervical dislocation, frozen, and stored at −80°C until further use. Membranes were prepared by homogenizing brain samples in an ice-cold 0.25 M sucrose solution (10 vol) (mL/g wet tissue weight). The obtained suspensions were then centrifuged at 2,500 *g* for 10 min. Supernatants were collected and diluted 10 times in buffer containing 50 mM Tris-HCl (pH 7.4), 3 mM MgCl_2_, 100 mM NaCl, and 0.2 mM EGTA, and then centrifuged at 23,000 *g* for 30 min. The pellets were homogenized in 800 μL of an ice-cold sucrose solution (0.32 M), aliquoted, and kept at −80°C. For [^35^S] GTPγS binding assays, 2 ug of protein were used per well. Samples were incubated with and without the test compound for 1 h at 25°C in an assay buffer containing 30 mM GDP and 0.1 nM [^35^S] GTPγS. Bound radioactivity was quantified using a liquid scintillation counter. Non-specific binding was defined as binding in the presence of 10 μM GTPγS; basal binding refers to binding in the absence of the agonist. The data were expressed as a mean percentage of activation above the basal binding. [S^35^] GTPγS binding by DAMGO and Tianeptine was plotted, with the x-axis representing concentration and the y-axis representing the percentage of activation against the background. EC_50_ values were calculated using GraphPad Prism software. The *E*_max_ is expressed as a percentage of activation above basal binding, which is set at 100%, and the basal binding refers to binding in the absence of the agonist.

### Tianeptine-induced analgesia

The tail immersion test (TI) consisted of immersing the tail of mice in a bath containing hot water (52°C) and then measuring tail withdrawal latency. A cut-off of 10 s was used to cease the test if the mice did not remove their tail.

The hot plate (HP) test consisted of measuring the latency to jump from a 52°C-heated surface with a cut-off of 300 s. The percentage of the maximum possible effect was calculated as the tail withdrawal or jump latencies divided by the cut-off period and multiplied by 100 [e.g., (14)].

To study the effect of cumulative doses of tianeptine-induced analgesia in MOR+/+ and MOR–/– mice (0, 2, 4, 8, 16, and 20 mg/kg, s.c., in ascending order), tianeptine (National Institute of Mental Health) dissolved in a 0.9% NaCl solution and administered intraperitoneally in a volume of 10 ml/kg, or saline, was injected. TI was done 45 min after the injection. The next dose was injected directly after the test, and a new TI was realized 45 min later. This was repeated until the last dose. A hot plate test was done just after the last TI.

To study the kinetics of the analgesic effects of 2- and 20-mg/kg doses of tianeptine in MOR+/+ and MOR–/– mice, tianeptine or saline was injected, and TI was done repeatedly every 5 min until 1 h after drug injection (13 tests/mouse). A hot plate test was done just after the last TI test.

For the tolerance study, we used methodological parameters to measure that acute tianeptine-induced analgesia fully recruited MOR (52°C-hot water bath, 20-mg/kg tianeptine dose). Commercial mice were treated chronically with tianeptine (i.p., twice daily, at least 8 h apart). TIs were done just before (for baseline) and 45 min after the AM injection. This was repeated 9 days later, as in Darcq et al. (15).

### Tianeptine-induced hyperlocomotor activity

Animal locomotor activity was monitored using a VersaMax System with X-Y sensors. We first measured the acute effect of cumulative subcutaneous doses of tianeptine (0, 4, 8, 16, 32, and 64 mg/kg, s.c., in ascending order) on hyperlocomotor activity. Then, the chronic effect of tianeptine on locomotor activity response was studied in c57bl/6:sv129 commercial mice, where tianeptine was injected intraperitoneally to study tolerance to tianeptine-induced analgesia. The mice were placed in locomotor activity boxes for a 30-min baseline recording without any injection. Then tianeptine or saline was injected i.p., and locomotor activity was registered for the next 2 h. Tianeptine doses (0, 10, and 30 mg/kg) were selected based on **Figure 3A** results and were injected on days 1, 4, 8, 11, and 15 based on Darcq et al. (15). The mice that received tianeptine at the highest dose were also injected with this same dose on days 16 and 17.

### Conditioned place preference

Following 5 days of handling and habituation to saline injection, the mice were tested in the conditioning place-preference paradigm (16) to evaluate the rewarding properties of tianeptine. The apparatus consisted of two Plexiglas chambers with different shape patterns and floors separated by a central corridor with sliding doors connecting the alley with the chambers (PanLab, Harvard apparatus, Spain). The location of the mice was recorded using weight detectors located throughout the apparatus. During the preconditioning phase, naive mice were placed in the corridor and had free access to both compartments for 20 min, with the time spent in each compartment recorded. Treatments were further counterbalanced between compartments to use an unbiased procedure. No initial place aversion for the different compartments was observed in the experiment. The conditioning phase lasted 3 days with two daily conditioning sessions. The mice were injected morning and afternoon with either saline or tianeptine (30 mg/kg) and confined in the corresponding paired chamber for 45 min. The mice in the control group received saline injections in the morning and the afternoon, followed by a 45 min confinement. The testing phase was conducted on the fifth day in a drug-free state with free access to both chambers for 20 min, and time spent in each chamber was recorded. The data are expressed as the time spent in each chamber.

### Statistics

3-way RM-ANOVAs were used to analyze tianeptine-induced increased latency of tail withdrawal in the tail immersion tests (treatment × genotype × dose for the effect of cumulative doses of tianeptine; treatment × genotype × time for the temporal analgesic effect studies; dose and time as within-subject variables). *T*-tests were used to analyze tianeptine-induced latency to jump in the hot plate tests. A 2-way RM ANOVA was used to analyze tolerance to tianeptine-induced analgesic effects (treatment × day, the latter as a within-subject variable). Two-way RM ANOVAs were used to analyze the tianeptine-induced hyperlocomotor effect (genotype × dose, the latter as a within-subject variable; treatment × time, the latter as a within-subject variable) and tolerance to this effect (treatment × day, the latter as a within-subject variable). One-way RM ANOVAs were used to analyze 30-mg/kg tianeptine-induced hyperlocomotion on days 16 and 17. Finally, 2-way RM ANOVAs were used to analyze Tianeptine-induced conditioned place preference in MOR+/+ mice and in MOR–/– mice (Test × Compartment, both as within-subject variables).

## Results

### Tianeptine induces G protein activation via MOR

**Figure 1:**
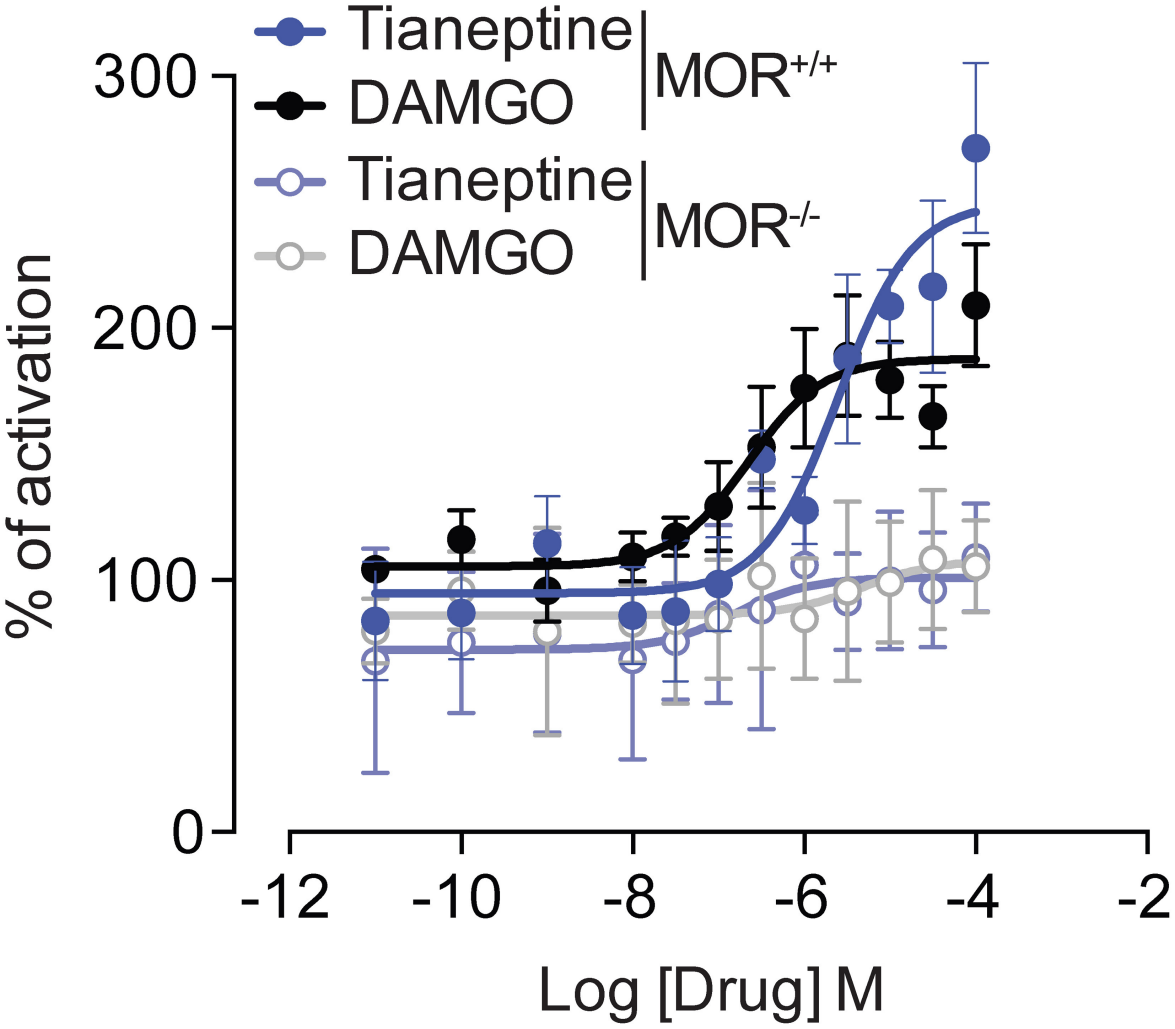
Tianeptine-stimulated [35S]GTPγS binding requires MOR. [35S] GTPγS binding of Tianeptine and DAMGO in MOR+/+ mouse brain membranes vs. MOR–/– mouse membranes. The data are the means of triplicate measurements, with the standard deviation shown as error bars and are representative of two independent experiments.

To study the effect of tianeptine on MOR-induced signaling, we measured G protein activation by two MOR agonists, tianeptine and DAMGO, in the [S^35^] GTPγS binding assay using whole brain membrane preparations from MOR+/+ mice and MOR–/– mice (Figure 1). Tianeptine and DAMGO enhanced [S^35^] GTPγS binding activity similarly, with EC50 values of 4.7 μM (tianeptine) and 1.1 μM (DAMGO) in MOR+/+ and Emax values of 250% (tianeptine) and 193% (DAMGO) in MOR+/+. Tianeptine and DAMGO were inactive in membranes prepared from MOR–/– mice, demonstrating that these compounds require MOR activity.

### Tianeptine induces analgesia via MOR

**Figure 2:**
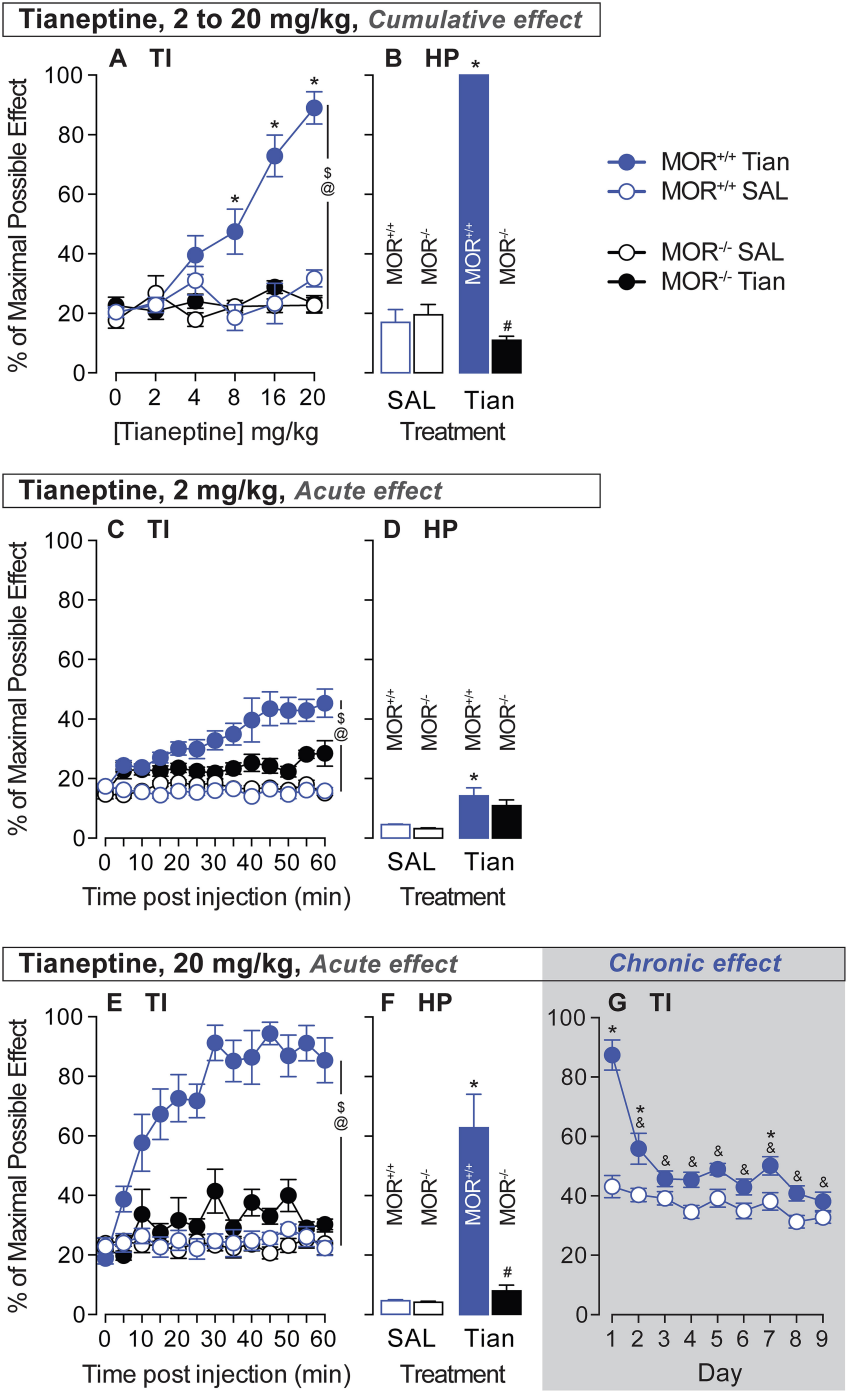
Tianeptine-induced analgesic effect is MOR-dependent, and chronic administration of tianeptine induces tolerance to this effect. Acute analgesic responses to tianeptine were analyzed using tail immersion (TI) tests and hot plate (HP) tests after injections of cumulative doses [0, 2, 4, 8, 16, and 20 mg/kg (s.c.) in ascending order, 45 min after each injection for TI and 50 min after the last injection for the HP, **A, B**], a low 2-mg/kg dose **(C, D)**, and a high 20-mg/kg dose **(E–G)**. Tolerance to analgesic responses to tianeptine was analyzed over 9 days of treatment (20 mg/kg, twice daily, **G**). ^$^*P's* < 0.05, the main effect of treatment. ^@^*P's* < 0.05, treatment x genotype x dose or treatment × genotype × time interaction effect. *P's < 0.05 vs. SAL mice. ^&^*P's* < 0.05, vs. day 1. *N*'s = 4–15/group. ^#^*P's* < 0.05 vs. MOR +/+ tian mice.

To test the analgesic effect of tianeptine and the MOR-specificity of this effect, we tested the analgesia induced by tianeptine using a tail immersion test in MOR+/+ and MOR–/– mice. Tianeptine effects on tail withdrawal latencies depended upon the dose and the genotype (treatment × dose × genotype interaction effect, 52°C bath, *F*_5,140_ = 5.78, *p* < 0.0001; Figure 2A). Importantly, there was no tianeptine-induced analgesia effect in MOR–/– mice (main effect of treatment in MOR–/– only, *P's* > 0.05; Figure 2A), indicating that tianeptine-induced analgesia is mediated by MORs. The tianeptine-induced analgesic effect was statistically significant at the 8, 16, and 20-mg/kg doses in MOR+/+ mice (main effect of treatment in MOR+/+ only, *P's* < 0.01; Bonferroni's multiple comparison tests vs. saline, *P's* < 0.05 at 8-, 16-, and 20-mg/kg doses, *P's* > 0.05; Figure 2A). The analgesic effects that we observed here are due to the sum of each dose previously administered, as behavioral measures are taken every 45 min. Immediately at the end of the last tail immersion measure (20 mg/kg), the mice were placed on a hot plate (HP) at 52°C. Cumulative doses (2–20 mg/kg) of tianeptine were also effective at increasing the latency to jump in a hot plate test (SAL vs. tianeptine in MOR+/+ mice, *t*_14_ = 36.32, *p* < 0.0001; Figure 2B), and this effect was MOR-dependent (tianeptine-treated MOR+/+ vs. tianeptine-treated MOR–/– mice, *t*_22_ = 64.51, *p* < 0.0001; Figure 2B). These data demonstrate that tianeptine induces an analgesic effect via MOR.

Then, we tested the kinetics of the analgesic effect of a low and high dose of tianeptine to determine the duration needed to obtain tianeptine's maximum analgesic effect. In a 52°C water bath, tail withdrawal latencies increased after 2-mg/kg tianeptine injection (s.c.), and under these conditions, tianeptine-induced analgesia depended upon both the genotype and the time of the test (Treatment × Genotype × Time interaction effect, *F*_12,336_ = 2.49; *p* = 0.004; Figure 2C). A low 2-mg/kg dose of tianeptine was also effective at increasing the latency to jump in the hot plate test (SAL vs. tianeptine in MOR+/+ mice, *t*_14_ = 2.77, *p* = 0.02; Figure 2D). A high 20-mg/kg dose of tianeptine induced an analgesic effect that was time- and genotype-dependent (treatment × genotype × time interaction effect, Figure 2E; 52°C, *F*_12,336_ = 4.79; all *P*'s <0.001). Acute 20-mg/kg dose of tianeptine was ineffective in increasing tail withdrawal latencies in a 52°C water bath in MOR–/– mice (main effect of treatment in MOR–/– mice; *F*_1,14_ = 4.26, *p* > 0.05; Figure 2G). Using a hot plate procedure, the latency to jump from the heating surface was increased in the mice that were previously injected with 20-mg/kg of tianeptine (SAL vs. tianeptine in MOR+/+ mice, *t*_14_ = 3.94, *p* = 0.002; Figure 2F), and this analgesic effect was MOR-dependent (tianeptine-treated MOR+/+ vs. tianeptine-treated MOR–/– mice, *t*_18_ = 4.8, *p* = 0.0001; Figure 2F). The tianeptine effect lasts at least 1 h after tianeptine administration (for low and high doses, see Figures 2C, E). Altogether, these results demonstrate that the tianeptine-induced analgesic effect is mediated by MOR.

### Chronic tianeptine induces tolerance to analgesia

We then tested whether chronic administration of tianeptine induces tolerance to the analgesic effect. The highly effective dose of 20 mg/kg was used to test whether tolerance to tianeptine-induced analgesia develops in MOR+/+ mice only (commercial mice, c57bl/6:sv129). The 20-mg/kg tianeptine ability to increase tail withdrawal latency in a 52°C water bath (main effect of treatment, *F*_1,27_ = 59.94, *p* < 0.0001; Figure 2G) decreased over time (main effect of time, *F*_8,216_ = 19.45, *p* < 0.0001; treatment × time interaction effect, *F*_8,216_ = 8.48, *p* < 0.0001; Bonferroni's multiple comparison tests vs. day 1 in tianeptine-treated mice, all *P's* < 0.0001; Figure 2G) and became ineffective statistically significant from day 3 of treatment (Bonferroni's multiple comparison tests vs. saline, *P's* < 0.05 at days 1, 2, and 7, all other *P's* > 0.05; Figure 2G). These data demonstrate that chronic treatment induces tolerance to tianeptine-induced analgesia.

### Chronic tianeptine induces tolerance to hyperlocomotor activity

**Figure 3:**
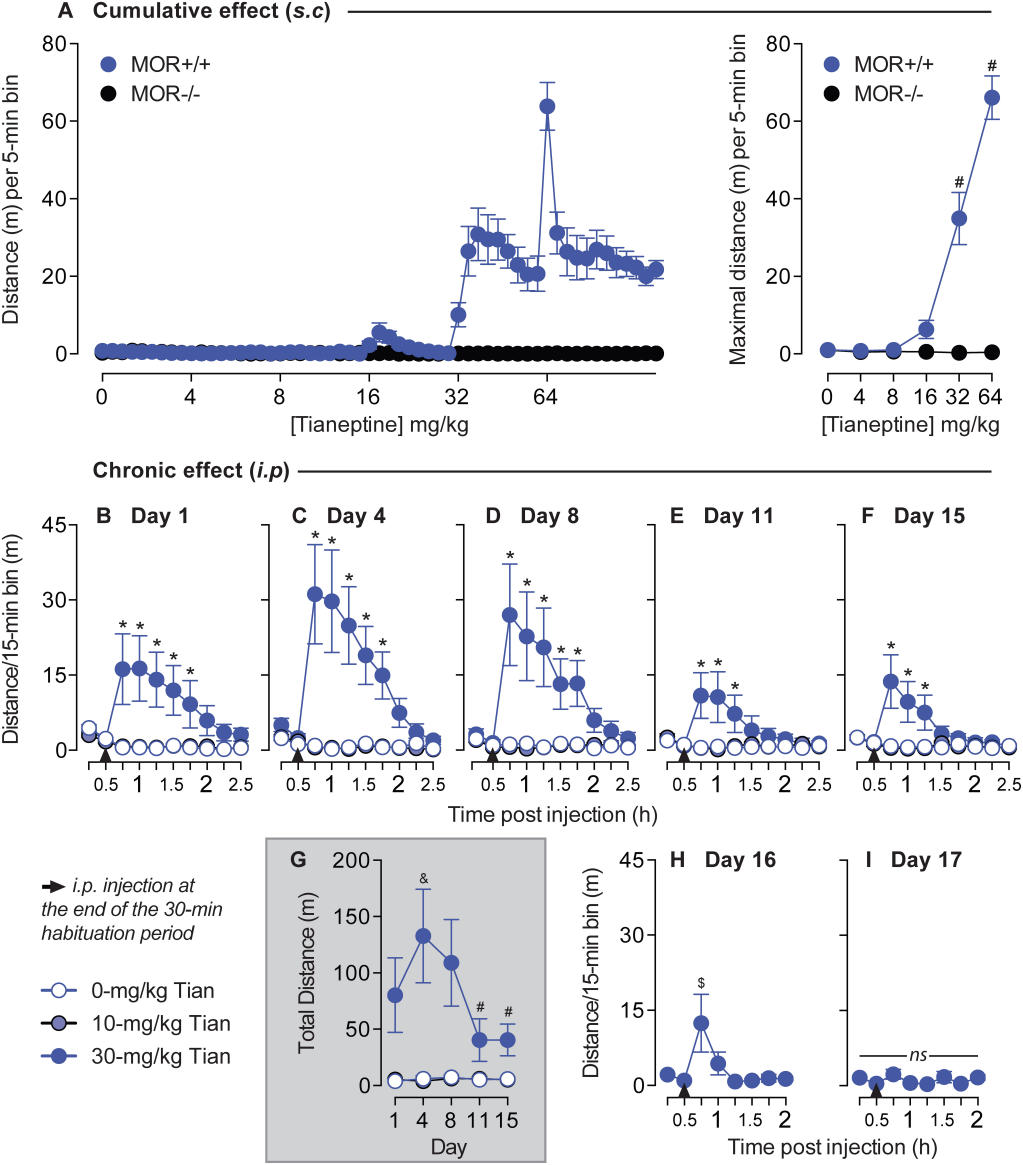
Tianeptine-induced hyperlocomotor effect is MOR-dependent, and chronic administration of tianeptine induces tolerance to this effect. **(A)** Cumulative doses of tianeptine-induced acute hyperlocomotion are MOR-dependent. Tianeptine (0, 4, 8, 16, 32, and 64 mg/kg, in ascending order, subcutaneous injections) dose-dependently increased locomotor activity [left: representation of the distance (m) per 5-min bin, and right: average of the maximal distance (m) per 5-min bin]. *P's < 0.0001, vs. 0-mg/kg dose. *N*'s = 10/group. **(B–I)** Mice were placed on locomotor activity boxes for 30 min for habituation, then tianeptine (0, 10, and 30 mg/kg, i.p.) was injected, and locomotor activity was recorded for 2 h on day 1 **(B)**, day 4 **(C)**, day 8 **(D)**, day 11 **(E)**, and day 15 **(F)**. **(G)** Shows the total distance after tianeptine or saline injection over days. 30-mg/kg tianeptine-treated mice received extra sessions on days 16 **(H)** and 17 **(I)**. *P's < 0.05 vs. SAL mice. ^&^*p* < 0.05, vs. Day 1. ^#^*P's* < 0.05, vs. days 4 and 8. ^$^*p* < 0.05, vs. the first timepoint of the habituation period (0–15 min). *N*'s = 8/group.

As morphine-induced hyperlocomotion is well established (15) and the anatomical structures and neurotransmitter systems that drive locomotor activity overlap those that mediate positive reinforcement and reward (17), we examined the effects of tianeptine on locomotor behaviors. We tested whether tianeptine induced a hyperlocomotor effect by injecting cumulative doses of tianeptine in both MOR+/+ and MOR–/– mice. Tianeptine doses (0, 4, 8, 16, 32, and 64 mg/kg) were administered subcutaneously in ascending order in MOR+/+ and MOR–/– (Figure 3A), and locomotion was measured for 1 h in the chamber. Tianeptine at 32 and 64 mg/kg doses produced hyperlocomotion in MOR+/+ mice but not in MOR–/– (Main effect of escalating tianeptine Dose, *F*_5,90_ = 61.72, *p* < 0.0001; Genotype × Dose interaction effect, *F*_5,90_ = 63.02, *p* < 0.0001; Bonferroni's multiple comparison tests vs. Tianeptine 0 mg/kg in MOR+/+ mice, *P's* < 0.0001 at 32 and 64 mg/kg doses; all *P's* > 0.05 in MOR–/– mice; Figure 3A, maximal distance) indicating that the hyperlocomotor effect induced by tianeptine is MOR-dependent.

Next, we tested whether the tianeptine-induced hyperlocomotor effect would be modified after chronic administration in c57bl/6:sv129 commercial mice. Based on the previous experiment, we selected 10 and 30 mg/kg doses (Figure 3A). 30-mg/kg dose but not 10-mg/kg dose of tianeptine induces hyperlocomotor activity on day 1 main effect of treatment, *F*_2,21_ = 4.9, *p* = 0.02; main effect of time, *F*_9,189_ = 3.59, *p* = 0.0004; treatment × time interaction effect, *F*_18,189_ = 4.99, *p* < 0.0001; Bonferroni's multiple comparison tests vs. saline, *P's* < 0.05 at Times 15, 30, 45, 60, and 75 min post 30-mg/kg tianeptine injection, all other *P's* > 0.05; Figure 3B), day 4 (main effect of treatment, *F*_2,21_ = 9.39, *p* = 0.001; Main effect of Time, *F*_9,189_ = 7.28, *p* < 0.0001; treatment × time interaction effect, *F*_18,189_ = 8.24, *p* < 0.0001; Bonferroni's multiple comparison tests vs. saline, *P's* < 0.05 at Times 15, 30, 45, 60, and 75 min post 30-mg/kg tianeptine injection, all other *P's* > 0.05; Figure 3C), day 8 (main effect of treatment, *F*_2,21_ = 6.77, *p* = 0.005; Main effect of Time, *F*_9,189_ = 5.36, *p* < 0.0001; treatment × time interaction effect, *F*_18,189_ = 5.65, *p* < 0.0001; Bonferroni's multiple comparison tests vs. saline, *P's* < 0.05 at Times 15, 30, 45, 60, and 75 min post 30-mg/kg tianeptine injection, all other *P's* > 0.05; Figure 3D), day 11 (main effect of treatment, *F*_2,21_ = 3.26, *p* = 0.058; main effect of time, *F*_9,189_ = 2.69, *p* = 0.006; treatment × time interaction effect, *F*_18,189_ = 3.91, *p* < 0.0001; Bonferroni's multiple comparison tests vs. saline, *P's* < 0.05 at Times 15, 30, and 45 min post 30-mg/kg tianeptine injection, all other *P's* > 0.05; Figure 3E) and day 15 (main effect of treatment, *F*_2,21_ = 5.78, *p* = 0.01; Main effect of Time, *F*_9,189_ = 3.52, *p* = 0.0005; treatment × time interaction effect, *F*_18,189_ = 4.46, *p* < 0.0001; Bonferroni's multiple comparison tests vs. saline, *P's* < 0.05 at times 15, 30, and 45 min post 30-mg/kg tianeptine injection, all other *P's* > 0.05; Figure 3F). The temporal hyperlocomotor effect of 30-mg/kg tianeptine lasted for 75 min on days 1, 4, and 8 but started to be shorter on days 11 and 15, where it lasted no more than 45 min post-injection (Figures 3B–F).

Repeated injections of 30-mg/kg tianeptine changed hyperlocomotor activity responses over days (main effect of treatment, *F*_2,21_ = 9.02, *p* = 0.002; main effect of day, *F*_4,84_ = 3.85, *p* = 0.006; treatment × day interaction effect, *F*_8,84_ = 3.84, *p* = 0.0007; Figure 3G). Locomotor activity first increased from day 1 to 4 as originally expected (Bonferroni's multiple comparison tests vs. day 1, *p* = 0.03, all other *P's* > 0.05; Figure 3G) but decreased afterward on days 11 and 15 (Bonferroni's multiple comparison tests vs. days 4 and 8, *P's* < 0.01, all other *P's* > 0.05; Figure 3G). Based on these results, 30-mg/kg tianeptine-treated mice were again injected with this same dose on days 16 and 17, and locomotor activity was recorded for 90 min post-injection (as this has previously been determined as the maximal temporal window where tianeptine could exert its effect on hyperlocomotion). 30-mg/kg tianeptine still increased locomotor activity on day 16 (*F*_7,49_ = 3.75, *p* = 0.003; Figure 3H), but the effect was even shorter than previously measured (Bonferroni's multiple comparison tests vs. the first 15-min habituation period, *p* = 0.006 at the 15-min timepoint post-injection, all other *P's* > 0.05; Figure 3H). There was no more increased locomotor activity induced by 30 mg/kg of tianeptine on day 17 (*F*_7,49_ = 2.02, *p* = 0.07; Figure 3I), suggesting the development of a tolerance to the tianeptine-induced hyperlocomotion effect using this paradigm.

### Tianeptine induces conditioned place preference via MOR

**Figure 4:**
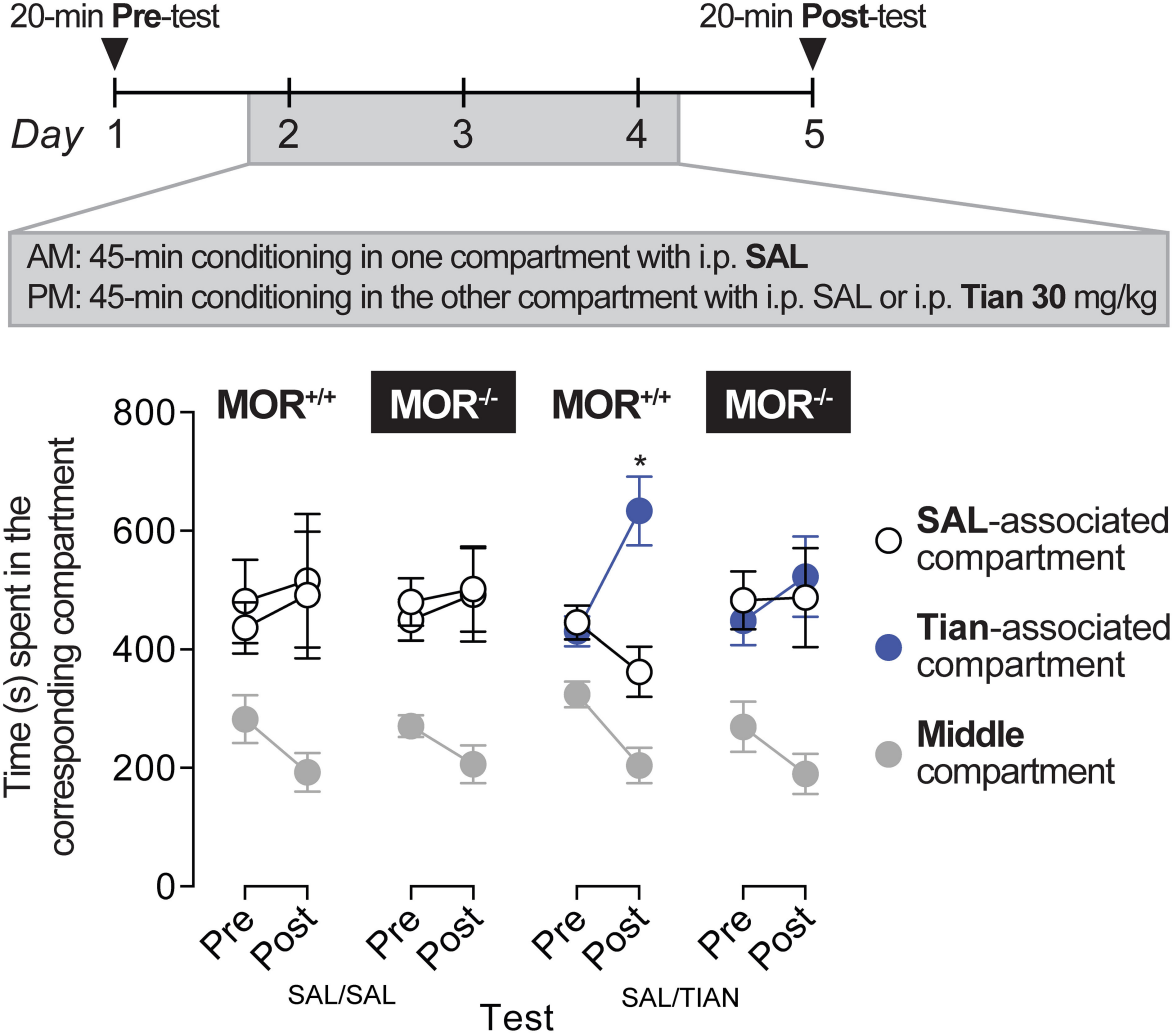
Tianeptine (30 mg/kg) induced a conditioned place preference, and this effect was MOR-dependent. MOR+/+ and MOR–/– mice were allowed to freely explore two compartments for 20 min on days 1 and 5. The two compartments differed in their texture, color, and spatial configuration. On days 2, 3, and 4, mice were injected (i) with saline in the mornings and confined in one of the two compartments for 45 min and (ii) with 30 mg/kg of tianeptine or with saline for controls in the afternoons and confined in the other compartment again for 45 min. MOR+/+ mice that received tianeptine in the afternoons next preferred the tianeptine-associated compartment compared to the saline-associated compartment (in tianeptine-treated MOR+/+ mice, Main effect of Test, *F*_1,9_ = 10.27, *p* = 0.01; Main effect of Compartment, *F*_1,9_ = 5.88, *p* = 0.04; Test × Compartment interaction effect, *F*_1,9_ = 6.55, *p* = 0.03; Bonferroni's multiple comparison tests vs. saline-associated vs. tianeptine-associated compartment, *p* = 0.01 on day 5). Tianeptine did not promote a conditioned place preference in MOR–/– mice. *p = 0.01, vs. a saline-associated compartment. *N*'s = 6–10/group.

The potential rewarding effect of 30 mg/kg of tianeptine was tested after three consecutive conditioning trials, as described previously (16, 18). Tianeptine (30 mg/kg) produced a statistically significant preference for the side paired with the drug in MOR+/+ mice (Figure 4; main effect of the test, *F*_1,9_ = 10.27, *p* = 0.01; main effect of the compartment, *F*_1,9_ = 5.88, *p* = 0.04; test × compartment interaction effect, *F*_1,9_ = 6.55, *p* = 0.03). Importantly, tianeptine did not produce a conditioned place preference in MOR–/– mice, suggesting that the rewarding effect of tianeptine is mediated by MORs.

## Discussion

Previous research identified tianeptine as a mu-opioid receptor (MOR) agonist. Our results are in line with these findings. Indeed, using the [S^35^] GTPγS assay, we found that tianeptine signaling involves MOR in the brain, and this MOR-recruiting effect is comparable to that of DAMGO, a traditional MOR agonist. Our data show that MORs are required for the analgesic, locomotor, and rewarding effects of tianeptine, which are present in MOR+/+ but not in MOR–/– mice. Also, we show that repeated administration of tianeptine led to decreased analgesic and hyperlocomotor responses, suggesting the development of tolerance to tianeptine in MORs. These results indicate that the opioid-like behavioral effects of tianeptine necessitate MOR and that tolerance can develop after repeated administration.

Tianeptine induces a strong analgesic effect in control animals but not in MOR–/– mice (Figure 2). This is not surprising given that tianeptine has been characterized as a full MOR agonist (7), and tianeptine reduction of depressive-like behaviors was abolished in MOR–/– mice. Our data show that a high dose of tianeptine (20 mg/kg) could also induce some analgesia in the MOR–/– mice (tail immersion test, Figure 2C, and hot plate test, Figure 2F) but to a smaller extent than in MOR wildtype mice. It has been shown that tianeptine also binds to delta opioid receptors (DOR) (7). Given the implication of DOR in pain control (19), it is tempting to hypothesize that the tianeptine-induced analgesic effect in MOR–/– mice could be the result of DOR activation.

We demonstrated a long-lasting acute analgesic effect induced by tianeptine that can be present until 1 h after the injection (Figure 2). This is in contrast with another study showing that the acute analgesic effect of tianeptine was optimal 15 min after drug injection but disappeared after 1 h (8). These pharmacodynamic differences of tianeptine could result from the use of different mouse strains: c57bl/6 or c57bl/6:sv129 (50:50), or also from the use of different behavioral tests (in 8: HP and in our study: TI). However, in both studies, the maximal effect of hyperlocomotor activity induced by tianeptine appeared 15 min after the injection [(8); Figure 3 of the present study], suggesting that the tianeptine pharmacokinetic profile should be the same in the two mouse strains. Another main difference between the two studies is that Samuels et al. (8) showed no tianeptine-induced analgesic tolerance, while we measured strong tolerance that occurred on the second day of treatment (Figure 2). Methodological differences could explain these discrepancies: the analgesic test used, the regimen and dosage of the chronic tianeptine treatment, the time when the analgesic test was done after tianeptine injection [30 mg/kg, twice a day for over 30 days, and acute administration of tianeptine 15 min before a hot plate test in Samuels et al. (8) vs. 20 mg/kg, twice a day for 9 days, with daily tail immersion tests before and 45 min after the AM-tianeptine injection in the present study].

An interesting result from this study is that despite inducing gradual psychomotor sensitization, as originally predicted from morphine data (15), we found tolerance to the hyperlocomotor effects of 30-mg/kg tianeptine (Figure 3). Indeed, our results show that tianeptine-induced hyperlocomotor activity on day 1 appeared to sensitize on day 4, after which the effect diminished until it completely disappeared on day 17 (7th tianeptine injection). Psychomotor sensitization refers to the progressive increase of locomotor activity after repeated exposure to the same dose of the drug, and this effect can be associated with the development of drug addiction (20). It is known that the development of psychomotor sensitization depends upon the intermittency of the treatment (21), and the protocol we used is known to induce psychomotor sensitization with repeated injections of 40 mg/kg of morphine (15). This lack of sustained psychomotor sensitization could explain the lower risk of abuse liability associated with tianeptine vs. other MOR agonists. Indeed, even if there are case reports of tianeptine abuse (22), the literature on this topic is scarce. However, such a hypothesis would necessitate being tested by directly comparing the self-administration of tianeptine vs. other MOR agonists in rodents.

As initially demonstrated by Samuels et al. (8), we showed that tianeptine induces a conditioned place preference that is MOR-dependent (Figure 4). We also showed that tianeptine-induced hyperlocomotor activity recruits MOR [(8); Figure 3], and the transient sensitization to this effect on the second day of injection could be the result of MOR activation. Thus, the proposed lower abuse liability of tianeptine compared to other MOR agonists is unlikely due to its inability to induce tolerance as originally hypothesized (8). It may be due to a lack of psychomotor sensitization and/or a lower intrinsic efficacy at MOR and thus be less likely to be abused. It is also important to note that while a supratherapeutic dose of tianeptine does not induce psychostimulant effects [no sedation, no euphoria, no dysphoria, no augmentation of energy (23)], tianeptine can actually be abused with higher doses (22), and this is probably due to its MOR agonist properties.

## Data availability statement

The raw data supporting the conclusions of this article will be made available by the authors, without undue reservation.

## Ethics statement

The animal study was reviewed and approved by the Canadian Council of Animal Care and the Animal Care Committees of McGill University/Douglas Mental Health University Institute approved all experimental procedures.

## Author contributions

FA, BK, and ED designed the experiments. FA, MM, and WM performed and analyzed the behavioral experiments. AE and FG performed the [S^35^] GTPγS assay. FA and ED wrote the manuscript. BK and AE edited the manuscript. All authors read and approved the submitted version.
